# Verification of quantitative analytical methods in medical laboratories

**DOI:** 10.5937/jomb0-24764

**Published:** 2021-06-05

**Authors:** Muhammad T. Abdel Ghafar, Muhammad I. El-Masry

**Affiliations:** 1 Tanta University, Faculty of Medicine, Departments of Clinical Pathology, Egypt; 2 Kafr El-sheikh University, Faculty of Medicine, Departments of Clinical Pathology, Egypt

**Keywords:** method verification, accreditation, quality, errors, verifikacija metoda, akreditacija, kvalitet, greške

## Abstract

**Background:**

Globally, all medical laboratories seeking accreditation should meet international quality standards to perform certain specific tests. Quality management program provides disciplines targeted to ensure that quality standards have been implemented by a laboratory in order to generate correct results. The hallmark of the accreditation process is method verification and quality assurance. Before introducing a new method in your laboratory, it is important to assess certain performance characteristics that reflect the concept of method verification.

**Methods:**

In this review, we illustrated how to verify the performance characteristics of a new method according to the recent guidelines. It includes an assessment of precision, trueness, analytical sensitivity, detection limits, analytical specificity, interference, measuring range, linearity, and measurement uncertainty.

**Conclusions:**

Although the presence of several updated guidelines used to determine the performance characteristics of new methods in clinical chemistry laboratories, the real practice raised several concerns with the application of these guidelines which in need for further consideration in the upcoming updates of these guidelines.

## Abstract (sr)

**Uvod:**

Sve medicinske laboratorije koje traže akreditaciju treba da ispunjavaju međunarodne standarde kvaliteta kako bi mogle da sprovode određene specifične testove. Program upravljanja kvalitetom pruža jasne smernice koje imaju za cilj da se obezbedi da laboratorija primenjuje standarde kvaliteta u cilju tačnosti rezultata. Osnova procesa akreditacije je verifikacija metoda i obezbeđivanje kvaliteta. Važno je da pre uvođenja nove metode u vašu laboratoriju procenite određene karakteristike performansi koje odražavaju koncept metode verifikacije.

**Metode:**

U ovom pregledu smo ilustrovali kako sprovesti proveru karakteristika performansi nove metode prema nedavnim smernicama. To uključuje procenu preciznosti, istinitosti, analitičke osetljivosti, granice detekcije, analitičku specifičnost, interferencije, opseg merenja, linearnost i mernu nesigurnost.

**Zaključak:**

Iako postoji nekoliko ažuriranih smernica koje se koriste za utvrđivanje karakteristika performansi novih metoda u laboratorijama za kliničku hemiju, stvarna praksa ukazuje na nekoliko problema u primeni ovih smernica koje je potrebno dodatno razmotriti u narednim verzijama ovih smernica.

## Introduction

The hallmark of the health care process in a country is the correct diagnosis, risk factor assessment, effective prophylactic and curative handling of the diseases. The pathologists and laboratory personals contribute mainly to diagnosis, effective treatment and follow up of patients. To achieve this role, efficient implementation of the quality system should be assigned in every laboratory seeking accreditation [1]. Accreditation regarding the ISO/IEC 17025 for testing and calibration laboratories [2] and ISO 15189 for medical laboratories [2] considered any institution or program meets the standards of quality set to perform certain specific tests presented forth by a worldwide scale of accreditation bodies including International Laboratory Accreditation Cooperation (ILAC). It spans the managerial and technical capabilities of a laboratory. The hallmark of the process of accreditation is method validation, verification and quality assurance [3].

Quality is a maintained process aiming for the appropriate performance of the tests from the first time and every time. Quality assurance system is the whole framework incorporating the inside and outside laboratory activities with appropriate laboratory practice and enhanced management skills to ensure that the correct assay performed on a correct sample obtained from right subjects at the correct well equipped place, generating perfect result interpreted precisely based on correct reference data. Implementation of quality concepts in the medical laboratories requires the presence of a quality management program targeted to ensure the reliability of the results generated by the laboratory [4].

Quality control is the process concerned with the control of performance errors in the analytical testing phase and verification of test results. Quality control can be assigned as an internal control that is performed by laboratories offering the day to day basis working quality assurance or external control performed consequently by many laboratories, and their results are statistically compared and evaluated for proficiency testing [4].

## Discussion

### Validation versus Verification concepts

According to International Vocabulary of Metrology 3 (VIM3), verification is defined as 'provision of objective evidence that a given item fulfils specified requirements' [5] whereas validation is 'verification, where the specified requirements are adequate for the intended use' [5]. In other words, validation is establishing the performance of a new diagnostic tool which is a manufacturer concern. However, verification is a process to determine performance characteristics before a test system is utilized for patient testing which is laboratory or user concern.

Once the method (reagents, procedure and the measure ment instrument) has been manufactured by a company, a proper method validation emerged and its results should be provided to the user. In this situation, a laboratory method validation is not required and method verification is more convenient [6].

There have been several publications discussing the guidelines of method validation and verification adopted by national and international organizations, regu lated and used by ISO 17025, ISO 15189 or by the Clinical Laboratory Improvement Amendments of 1988 (CLIA 88) [7] either generally [7] [8] [9] [10] [11] [12] [13] [14] [15] or specifically in the analytical chemistry [16] [17], toxicology [18], chemical pathology [19], food and drugs industry [20] [21].

### Errors in measurements

The main purpose of method validation and verification is error assessment – what is the scope of possible errors within your laboratory assay results, and to what extent this degree of errors could affect clinical interpretations and, consequently, patient care.

### Random Error

**Figure 1:**
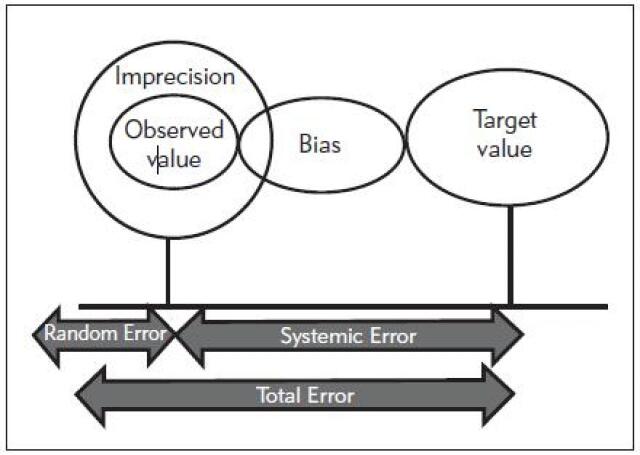
Total analytical errors in medical laboratories Note: the total error is the sum of both random and systematic errors which in turn represent the bias and imprecision values.

Random error is a type of measurement errors arising from the repeated assay. Hence it is considered a sort of imprecision issue and determined by the standard deviation (SD) and the coefficient of variation (CV) of test values (Figure 1). It is characterized by wide random dispersion of control values around the mean and exceeding both the upper and lower control limits [22]. It reflects problems affecting measuring techniques as noises, sample preparation as improper temperature stability [23]. Random error is calculated as the standard error of estimate (Sy/x) which is the SD of the points about the regression line. Sy/x represents the square root of the squared distance of results from the regression line divided by their numbers as in equation 1 (Table 1). The higher the Sy/x, the wider is the scatter and the higher is the amount of random error [24].

**Table 1:** Equations used for estimation of different verification parameters

	Parameter	Equation no	Equation	Remarks
1	Random error	1	\(Sy/x=\sqrt{\frac{\sum{(yi-Yi)^2}{\blacksquare\atop\blacksquare}}{(n-2)}}\)	Where: <br> Sy/x=Standard error of estimate<br> yi-Yi=The distance of each y-value from the regression line<br> n=number of y-values
2	Systematic error	2	\[Y = a+bX\] \[a=\frac{(\sum{y})(\sum{x^2})-(\sum{y})(\sum{xy})}{(n(\sum{x^2})-(\sum{x})^2}\] \[b=\frac{n(\sum{x}-(\sum{y})(\sum{y}))}{(n(\sum{x^2})-(\sum{x})^2}\]	Where: <br>*Y=Reference* *method values*<br> *X=Test method values <br> a=y-intercept<br> b=Slope of the regression line*
3	Interference	3	\(\textrm{Bias %} = \frac{\textrm{(concentration with interference - concentration without interference)}}{\textrm{(concentration without interferencex100)}}\textrm{X100}\)	
4	Trueness	4	\[\textrm{Verification interval}=\]\[X\pm2.821\sqrt{Sx^2+Sa^2}\]	Where:<br> X=mean of tested reference material<br> Sx=standard deviation of tested reference material<br> Sa=Uncertainty of assigned reference material (Manufacturer SD of IQC, Uncertainty of PT sample, Uncertainty of calibrator) 2.821 is the 99 per cent point of, t, of the t-distribution with 9 degrees (2n-1) degrees of freedom)
5	Precision	5A	\(S_r=\sqrt{\frac{\sum{(X_{di}-\overline{X}_d)^2}}{D(n-1)}}\)	Where: <br>Σ=summation <br> Sr=repeatability <br> D=total days number (5) <br> n=total replicates number per day (3) <br> di=replicates result per day (3 replicates)<br> xd=average of all results for day (d).
		5B	\(S_b=\sqrt{\frac{\sum{(X_d-\overline{X}_d)^2}}{D-1}}\)	where:<br>X_d_=average of all results for day d <br> X̄=average of all results.
		5C	\(S_t=\sqrt{\frac{n-1}{n}}(S_r^2+S_b^2)\)	Where: <br>S_t_=Total within lab precision <br> n = number of replicates per run (three).
6	Detection limits	6A	LOB = Mean blank+1.645 * SD blank	
		6B	LOD = Mean blank+3.3 * SD blank	
		6C	LOQ = Mean blank+10 * SD blank	
		6D	LOD = 3.3 σ/Slope	Where: <br> σ=the standard deviation of the response at low concentrations <br> Slope=the slope of the calibration curve.
		6E	LOQ =10 σ/Slope	
7	Error index	7	error index = (x-y)/TEa	Where: <br> TEa (total allowable error)
8	Uncertainty	8A	\(Us=\frac{(SD)L1^2+(SD)L2^2}{2}1/2\)	Where:<br> Us=Standard uncertainty, (SD) L1 and (SD) L2= the average SD of each control level, respectively, for the past 6 months.
		8B	UB = Test Result – Reference value	Where: <br>UB=Bias uncertainty
		8C	\[Uc = [(Us)2 + (uB)2]1/2\] \[Uc = [\frac{Us^2 + UB^2}{2}]1/2\]	Where: <br> Uc=the combined standard uncertainty
		8D	U = Uc x 1.96	Where: <br> U=the expanded uncertainty of the method, <br>1.96=coverage factor

## Systematic Error

**Figure 2:**
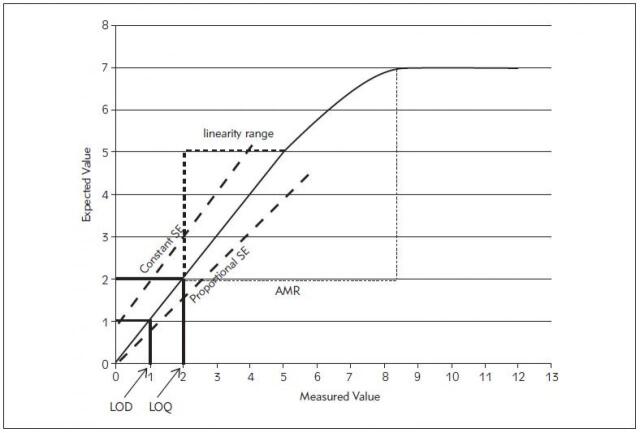
Point to point linear curve of the measured and expected values of a laboratory assay results of the serially diluted sample Note: it represents the limit of detection (LOD), the limit of quantitation (LOQ), linearity range, analytical measurement range (AMR), constant and proportional systematic errors (SE).

Systematic error reflects the inaccuracy problem in which the control observations are shifted in one direction of the mean and may exceeding one of the upper or lower limits. It is related mainly to the calibration problems such as impure, unstable calibration materials or improper standards preparation and inadequate calibration. In contrast to random errors, systematic errors could be evaded via correction of their causes [22]. Systematic errors could be proportional or constant (Figure 2). Systematic error is detected by linear regression analysis with y-intercept of the linear regression curve indicates the constant error while the slope indicates the proportional error as in equation 2 (Table 1) [24].

### Total Error Allowable (TEa)

TEa is the total error permitted by CLIA 88, based on the medical requirements, the available analytical methods and compatibility with proficiency testing (PT) expectations. The CLIA 88 have published allowable errors for a wide range of clinical and laboratory tests [25]. The original CLIA list of regulated analytes was based on medical practice in the past and missed several tests such as HbA1c or PSA that are used frequently at present. Recently, new proposed rules have been developed to expand the list of regulated analytes and define new criteria for acceptance limits to reflect the technological advancements and changes in the use of laboratory tests. The recent document proposed by Westgard JO and Westgard S, 2019 includes some tests deletions and many additions based on the PT availability, test significance, and feasibility of implementation. Several tests were added such as B-natriuretic peptide (BNP), hemoglobin A1c (HbA1c), prostate-specific antigen (PSA), troponin, C-reactive protein (highly sensitive), while other tests are deleted such as lactate dehydrogenase (LD) isoenzymes, quinidine, primidone, and procainamide. Moreover, the acceptance limits of other tests are changed from the target value (TV)±SD to TV±% such as alpha-fetoprotein, complement, immunoglobulins, human chorionic gonado tropin, thyroid-stimulating hormone (TSH) and others [26].

The allowable errors limits assigned by CLIA denote the maximum error allowable by federally mandated proficiency testing. These performance characteristics are currently used to investigate the acceptability of clinical chemistry analyzer performance [27] [28]. The total analytical error involves the sum of the random and systematic errors generated by the laboratory (Figure 1). The target of a quality assurance system is to maintain the total Analytical Error released by the laboratory below the total allowable error. Hence, the method should be modified or even rejected if the potential error generated by it exceeds the error allowable and could lead to misinterpretation [24]. It is possible to calculate the total errors using the precision and trueness protocols described in this literature by considering the confidence-interval criteria and single-value criteria in the evaluation of error [29] [30].

### Verification Outline Parameters

### Analytical Specificity: Interference studies

Analytical specificity is referred to the measurement capability of an analytical method to exclude any interference in general, either cross-reacting substances, matrix effects and other effects as heterophilic antibodies, and to detect only the measurand of interest [5]. Analytical specificity is verified using interference studies. The hemolysis, icterus, and lipaemia abbreviated as (HIL) together with the anticoagulants and preservatives as stated by Young [31] and Glick and Ryder [32] [33] to be the common interferents. They assessed the effect degree of interference produced by each interferent concentration on the analyte detection level by each of the chemistry instruments presented as »interferographs«.

The effect of 'HIL' on the analyte concentration can be avoided by using certain analytical systems that minimize haemoglobin, triglyceride, and bilirubin interference [34]. This can be achieved by using certain assay methods that are not liable to interference. Icterus, for instance, has been shown to have no significant effect on some phosphate assays (Ortho Vitros 250/950 and Hitachi 700/900 platforms) [35]. Some automated systems have a »HIL« index. However, it is not immune to interference itself. Samples containing extremely high bilirubin concentrations or gross haemolysis may produce non-specific lipemic flags. Moreover, raised lipemic index results may occur in samples containing high concentrations of immunoglobulins [36]. This interference may result in a false high HIL index which can be corrected by correlation of HIL index results with the sample results for bilirubin and triglyceride level. The available data with analyst experience play a role in identifying the interferences that are most likely to affect the measurement method [37].

The interference study is done by adding the tested interferent in a maximum concentration to the patient sample. With generating an effect, its concentration is lowered gradually till reached the concentration at which estimated analyte results considered valid. The bias % could be calculated using equation 3 (Table 1). This concentration cutoff of the interferent is thus determined and acceptable results could be generated if interferents are at levels below this [38]. Samples with interference in some meth ods for example, in the diagnosis of thyroid diseases due to the presence of heterophilic antibodies should be tested by other methods for estimation of the degree of interference [6].

### Analytical Sensitivity: Calibration curve, Detection limit

The sensitivity of a measurement system is the 'quotient of the change in an indication of a measuring system and the corresponding change in the value of a quantity being measured' [5]. In other words, analytical sensitivity is the possibility of detection of a low concentration and or change in concentration of an analyte in a biological specimen. Concisely, sensitivity is the function of detection limit studies and the slope of the calibration [6].

### Reportable Range (Analytical Measurement Range)

The reportable range is defined by CLIA [39] as »the span of test result values over which the laboratory can establish or verify the accuracy of the instrument or test system measurement response.« However, CAP defines the reportable range in the context of two distinct concepts; the analytical measurement range (AMR) and the reportable clinical range (CRR). AMR is the »range of analyte values that a method can directly measure on the specimen without any dilution, concentration, or other pretreatments not part of the usual assay process« [40]. The AMR is determined by the manufacturers, and should not exceed the linearity range as demonstrated between the estimated and actual analyte concentrations (Figure 2).

Verification of AMR is performed using matrix-appropriate materials which could be calibrators or commercial linearity standards via the linearity experiment [40]. They should be of low, mid, and high concentration covering the activity spectrum of the claimed AMR by the manufacturer or spanning from 50 to 150% of the target analyte concentration. Each of the samples with distinct concentrations should be assayed at least in duplicate to eliminate the imprecision effect. The measured values (on the X-axis) are displayed versus the assigned values (on the Y-axis) in point to point line graph for each analyte with the assessment of the slope, intercept and correlation coefficient data [41]. The reportable range should be within the manufacturer's AMR claims. The manufacturer's detection limit can be verified if the concentration of the highest calibrators or other used materials within the per cent TEa (10% to 15%) of the AMR upper limit and the lowest calibrator is within the minimum detectable level or approximating TEa per cent of the lower limit of the claimed AMR [42].

A clinically reportable range (CRR) is another proposed concept of the CLIA reportable range by CAP. It is similar to AMR but assumed to refer the wide range of analyte results, including those obtained with dilution or concentration of a patient sample. It should be applied for samples with analyte concentrations exceeding the AMR [43]. Each laboratory should verify the maximal amount of dilution is allowed to cover this range without affecting the assay accuracy and exceeding manufacturer's protocols for dilution. With implying this allowed dilution, samples giving an inaccurate assay result should be reported as exceeding the upper CRR limits.

### Accuracy and Trueness (Bias)

Accuracy is defined as the closeness of the agreement between the test result and accepted true value whereas, trueness is »closeness of agreement between the average of an infinite number of replicate measured quantity values and a reference quantity value« [5]. It can be evaluated as measure ment bias which is the quantitative »estimate of a systematic error« [5].

Several factors found to share in the production of measurement bias. One of these factors is the presence of analytical interferents as haemolysis, lipaemia and icterus or cross-reaction as heterophilic antibodies in immunoassays. Others involve the improper calibration matrix or faulty preparation. Also, poor sample processing, transport and preservation may have a role in measurement bias [6].

Measurement bias can be determined via comparing the sample results estimated by a certain method with certified reference materials values purchased from companies or organizations of high metro logical competence or participating in external quality control program that compare with consensus mean of estimated control values among the different laboratories using the same method or implying the interference study or assessing the recovery of the measurand in spiked natural samples [6].

The trueness can be assessed using two levels of quality control materials or certified reference materials as calibrators, or the proficiency testing material. If proficiency testing material used, the consensus mean and SD are used only if obtained from at least ten peer participants. Each analyte is measured in three replicates for 5 days with the calculation of mean and SD of the 15 measurements. Calculation of verification interval for the bias was performed using equation 4 (Table 1). If the verification interval includes the consensus peer group mean, trueness is accepted [44].

### Precision (Replication study)

The precision is a hallmark of the verification process. It is defined as the closeness of agreement between the measurands results or test values provided by multiple estimations in replicates under particular conditions which could be similar or changed [5]. These conditions could be the same method, by the same operator, on the same instrument, within the same laboratory, using the same reagent materials within a short period which represents the process entitled repeatability or over an extended period, but may include other conditions involving changes as new calibrations, calibrators, operators entitled as intermediate precision. Whereas, the previous process options but with different operators, on different instruments carried on different laboratories but with the same test reagents and the same test method was considered as reproducibility [45].

Accordingly, precision could be viewed at three levels; repeatability, intermediate precision and reproducibility. Or, precision could be regarded in accordance to the time component within the laboratory as repeatability (within run precision – intra-assay), between run precision (inter-assay), within-day precision, and between-day precision.

All over the entire precision estimation process, a single lot of reagents and calibrators should be used as using different lots could increase the observed variability. The material used for precision estimation should be stable, frozen pools of two or more concentration spanning the medical decision levels or measuring range of the instrument. Quality control material or pooled serum of minimum two concentration levels are the ideal materials for precision estimation [46].

For each of the provided levels of quality control materials or pooled sera, three replicates were measured per day (within run-repeatability-intra-assay), for five days (in between runs-intermediate precision). All of these replication results are collected and analyzed in the way to obtain the mean, standard deviation (SD) and coefficient of variations (CV) for each parameter level [44]. The within run repeatability standard deviation (S_r_) is calculated using the following equation 5A whereas inter-assay precision standard deviation (S_b_) is calculated using equation 5B and the total intra-laboratory precision standard deviation is calculated by equation 5C (Table 1) [44].

The standard deviations and CVs obtained for each analyte as estimated repeatability or within-laboratory precision should be statistically compared to the manufacturer's claim using analysis of variance (ANOVA) test of significance. The estimated within-laboratory or repeatability standard deviation should be less than the manufacturer's claim and hence demonstrated as precision consistent with the claim [44].

### Detection Limits

It is impossible for any reagent to detect zero concentrations of the analyte regardless of the reported measuring range of some reagents to have in their reagent package. It is necessary to present enough analyte concentration to be measured as an analytical signal not as »analytical noise,« that produced in the absence of analyte [16]. The lowest quantity of the analyte that could be reliably measured by the analytical methods is referred as the detection limit of the reagent. However, detection limits exist in three levels expressed as Limit of blank (LOB), Limit of Detection (LOD), and Limit of Quantification (LOQ) (Figure 2) [47].

Limit of Blank (LOB) is determined by EP17 as if repeated measures of the reagent blank devoid of any analyte were performed, and the highest reachable concentration confirmed [48]. The limit of blank should be determined if the repeated blank assay revealed different results. LoB is determined by repeated measurements of a blank sample and calculation of the mean and the standard deviation (SD) of the obtained results using equation 6A (Table 1) [49].

Limit of Detection (LOD) is the minimal quantity of the analyte in a sample which produces excess signals than those generated by the blank but not necessarily expected as a true value. Limit of Quantification (LOQ) is the minimal concentration by which the analyte could be reliably measured with particular acceptable bias and imprecision value, commonly CV=20%. It is also defined without bias inclusion as »Functional sensitivity« [33] [50].

Several methods have emerged for estimation of detection and quantitation limits. These methods include visual definition, the signal-to-noise ratio (with 2 or 3 times for LOD and 10 times for LOQ), the standard deviation of the blank and the calibration line at low concentrations [11].

Implying the standard deviation of the blank method, the blank is measured ten or more times with calculation of the mean and SD of the blank results. The limits are consequently calculated using equation 6B, 6C (Table 1)[51]. However, if the blank had no background noise, method implying the standard deviation of the response and the slope could be performed using a standard curve to determine LOD and LOQ [47]. Five concentration of calibrators at very low values close to zero are measured six or more times. The y-intercepts of regression lines represent the standard deviation of the response. Hence, the detection limits may be expressed following equations 6D, 6E (Table 1) [51].

### Verification of reference intervals

Reference interval is the most important element in the analysis process as it is the value by which the clinician could interpret the patient laboratory results. Most of the laboratories pay little attention to establishing the reference intervals or even verifying the reference interval provided by the manufacturers.

Reference interval typically represents the central 95% interval of the observed analyte values among the healthy subjects. The role of laboratories is just verifying the reference intervals claimed by the manufacturer and »transferring« them to the laboratory. »Establishment« of reference intervals is another issue.

The reference interval could be verified by the laboratory by collecting samples from 20 reference individuals, selected from the studied population, and comparing the obtained test results to those of the manufacturer [52]. The selection of the reference individual should obey certain exclusion criteria as (history of diseases, surgery, smoking, drugs, and conception) and partitioning criteria including age, gender, race, etc. [53]. Written informed consent should be signed by all of the reference subjects and the pre-analytical precautions (e.g., fasting state, physical exercise, medication), with specimen collection variables as timing, tourniquet application, tube selection, and sample processing as centrifugation, transport and preservation should be assigned [54].

If no more than two of the 20 tested subjects' values (or 10% of the test results) fall outside the manufacturers claimed interval, this reference interval is considered verified. If more than 2 values exceed the limits, the claimed interval could not be adopted and more data collection is emerged [52].

Establishment of reference interval has emerged if verification of the claimed interval has been not assigned. The non-parametric approach is the most commonly used method for establishing reference intervals due to its ease and applicable for any distribution nature [52]. In this method, 120 samples obtained from reference subjects and ranked by concentration order one simply puts the values obtained from reference individuals in rank order by concentration with rank 3 is the 2.5^th^ percentile; rank 118 is the 97.5^th^ percentile and ranks 1 and 7 define the 90% confidence interval of the 2.5^th^ percentile, and ranks 114 and 120 define the 90% confidence interval of the 97.5th percentile. The reference interval occupies the central 95% of the distribution and the 90% confidence limits on both endpoints [52].

### Comparability experiments

Comparability is the agreement between results obtained for an analyte using different measurement procedures (different methods or different instruments). As stated by Westgard et al. [30] and CLIA [55], it could be assessed using 40 to 100 samples assayed on both methods under examination (two field methods), or between one tested method and a reference method or on both instruments on the same day over 8 to 20 days (preferably within 4 hours), with specimens spanning the clinical range and representing a diversity of pathologic conditions. Daily analysis of two to five patient specimens should be followed for at least 8 days if 40 specimens are compared and for 20 days if 100 specimens are compared in replication studies [55].

Different methods could be used for the assessment of the proper comparability as Spearman coefficient of Correlation, Paired test for difference, linear regression as Deming regression or Passing-Bablok regression and Bland-Altman analysis.

Either a linearity regression analysis for the result obtained from the 2 instruments or methods and calculate the correlation coefficient »r« or error-index plot using equation 7 (Table 1) can be used to assess the acceptability of comparability. The test-method results (*y*-axis) are displayed versus the comparative method (*x*-axis) if the two methods correlate perfectly, the data pairs plotted as concentrations values from the reference method (*x*) versus the evaluation method (*y*) will produce a straight line, with a slope of 1.0, a y-intercept of 0, and a correlation coefficient (*r*) of 1 [56]. The results are considered to be comparable if no more than 10% of results' error-index exceed +1 or are less than -1 or the correlation coefficient »r« more than or equal 0.95 [57] [58] [59].

### Uncertainty

It is a parameter reported with the test results characterizes the probability interval concerning the true value around the laboratory result [60]. Hence, uncertainty offers a quantitative determination of the confidence range and the expected variability in a laboratory result when the test is performed on different instances. Consequently, both imprecision (SD) and inaccuracy (bias) are taken into account in the measurement uncertainty estimate. With negligible or corrected bias, the measurement uncertainty can be estimated using only the imprecision times a coverage factor (magnitude of the factor is based on the confidence level assigned to the result distribution). Thus, measurement uncertainty is adopted in standard deviation (SD) units, the coefficient of variation (% CV), confidence intervals (CI's) or ranges (R's) [61].

Two interchangeable approaches were employed for uncertainty estimation; the so-called »bottom-up« and »top-down« approach. The »bottom-up« approach as proposed by the Guide to the Expression of Uncertainty of Measurement (GUM) identify, quantify and incorporate each source or origin of measurement uncertainty in the assay process into a final estimate entitled expanded combined uncertainty using statistical measures [62] [63]. However, it is a complicated diverse approach applicable mainly for metrology institutions and accredited reference laboratories and challenging to be assigned by the medical laboratories [64]. Instead, the »top-down« approach can be easily applied by clinical laboratories using intralaboratory (for imprecision) and inter-laboratory (for bias estimation) quality control data [65] [66] [67].

The measurement uncertainty is estimated reliably via the collection of 180 measures over six months of at least two levels of a single lot of stabilized control materials [68] [69]. For new methods, a minimum of 30 replicate determinations of appropriate control is required to calculate the standard deviation (SD). If bias is significant or known, calculate the combined standard uncertainty using the following equations 8A, 8B, 8C, 8D (Table 1) [61].

As the usage of quality control samples may yield overestimated uncertainty values due to interference by matrix effects in stabilized control materials. So, split-sample techniques using patient samples could be more accurate and close to the real uncertainty estimates [70].

### Main concerns and future perspectives

Several guidelines have been applied to determine the performance characteristics of new methods in clinical chemistry laboratories. However, several questions have emerged with the application of these guidelines in real practice.

Assessment of method trueness as previously illustrated, is hindered because of the presence of a wide range of verification interval. It can be raised due to the presence of discrepant results on repeated measurement which confer a wide standard deviation that reflected by a wide verification interval. Thus the mean result of the measurand may fall within the verification interval due to the inappropriate wide difference of the measurand results on repeated measures not because of the closeness of measurand results to the true value which is the hallmark of trueness definition.

The latest version of CLSI (EP15) for precision assessment expresses the main defect in the assessment of only 3 samples in the run which confer some statistical bias. Even though this guideline has improved relative to the previous version (CLSI EP5 A2) which employed only duplicate measures for 20 days, however, statistical bias may still exist due to the small number of replicates in the run.

The main concern arises with the application of the previously described guidelines is inappropriate acceptance criteria for some verification parameters. Regarding the reference interval verification, the acceptance criteria need to be revised for the presence of no more than 10% outside the validated range as the distribution shape of the results should be also considered. The sample results should be distributed normally. Skewed or bimodal distribution should not be accepted as it confers the inappropriate assignment of selection, exclusion and partitioning criteria for the included subjects for reference interval verification experiment. The recommended percentage (no more than 10%) of results that are accepted to fall outside the range of the claimed reference interval should be distributed equally outside the upper and lower limit of the claimed reference interval. Moreover, to our best knowledge, no acceptance criteria have been proposed for measurement uncertainty up to now.

Because of the previously described concerns experienced with the assignment of the current guidelines for method verification, it will be necessary to address these concerns in the future version of the verification guidelines. We recommend employing a much greater number of replicates in each run in the precision experiment to overcome the statistical bias of low sample size. Also, the estimated standard error of the mean of the measurand can be used instead of the standard deviation in the calculation of verification interval for the method trueness experiment. Moreover, the acceptance criteria for measurement uncertainty of a method are the main question and needs an answer in the future guidelines. Employment of CLIA 88 as acceptance criteria for measurement uncertainty of a method may be valuable with the expanded uncertainty of a measurand should not exceed the accepted standard deviation proposed by CLIA 88 for this measurand.

## Conclusion

Several global workgroups exert much effort in establishing protocols and guidelines to rule the process of verification in medical laboratories. Method verification is the main step in the process of enhancing the quality of laboratory results and a cornerstone in accreditation. Despite the presence of several updated guidelines used to determine the performance characteristics of new methods in clinical chemistry laboratories. However, real practice raised several concerns with the application of these guidelines which need further consideration in the upcoming updates of these guidelines.

## Dodatak

### Acknowledgements

We would like to express our special appreciation for Prof Amal El-Bendary for her intimate support.

### Contributors

MTAG researched literature and conceived the study. MTAG and MIE wrote the first draft of the manuscript. MTAG reviewed and edited the manuscript and approved the final version of the manuscript.

### Funding

No funding was received.

### Ethical approval

Not applicable.

### Conflict of interest statement

The authors stated that they have no conflicts of interest regarding the publication of this article.

### List of abbreviations

ILAC, International Laboratory Accreditation Cooperation; VIM3, International Vocabulary of Metrology 3; CLIA 88, Clinical Laboratory Improvement Amendments of 1988; SD, Standard deviation; CV, Coefficient of variation; TEa, Total error allowable; BNP, B-natriuretic peptide; PSA, Prostate-specific antigen; LD, Lactate dehydrogenase; TV, Target value; TSH, Thyroid-stimulating hormone; HIL, haemolysis/icterus/lipaemia; AMR, Analytical measurement range; CRR, Clinical reportable range; LOB, Limit of blank; LOD, Limit of detection; LOQ, Limit of quantification.

## 
